# Pregnant women's perspectives on antenatal care utilization in Cameroon: a cross- sectional mixed-methods study

**DOI:** 10.3389/fgwh.2026.1688295

**Published:** 2026-04-15

**Authors:** Robert Tchounzou, Harisse Ntongamuah Zisuh, André Wambo Simo, Crysantus Yimlefac Nzometia, Théophile Nana Njamen, Gregory Edie Halle Ekane

**Affiliations:** Department of Obstetrics and Gynecology, Faculty of Health Sciences, University of Buea, Buea, Cameroon

**Keywords:** antenatal care, Cameroon, maternal health, mixed methods, quality of care, service utilization, sub-Saharan Africa, women's satisfaction

## Abstract

Antenatal care (ANC) is essential for improving maternal and fetal health outcomes, yet utilization remains suboptimal in Cameroon. This study explored pregnant women's perspectives on determinants of ANC utilization and perceived quality of care in the Buea and Limbe Health Districts, conceptualizing satisfaction both as an outcome of service quality and a contextual influence on continued utilization. An explanatory sequential mixed-methods design was employed, comprising a cross-sectional survey of 410 pregnant women attending public, private, and faith-based health facilities, followed by 20 in-depth interviews. Quantitative data were analyzed using descriptive statistics and multivariable logistic regression, while qualitative data were thematically analyzed and integrated through side-by- side comparison. Only 45.6% of participants achieved the World Health Organization–recommended minimum of eight ANC contacts. Adequate utilization was significantly associated with receiving care in public facilities, formal employment, lower parity, and timing of ANC initiation. The association between second-trimester initiation and adequate utilization reflects cumulative attendance patterns rather than optimal timing and should therefore be interpreted cautiously. Key barriers to ANC use included financial constraints, long waiting times, negative provider attitudes, and delayed disclosure of pregnancy. Although overall satisfaction with ANC services was relatively high, dissatisfaction persisted regarding waiting times, indirect costs, and the quality of health education. Qualitative findings highlighted the importance of respectful, nonjudgmental provider behavior, partner support, privacy during consultations, and clear communication in shaping women's engagement with ANC services. Improving ANC utilization and quality in Cameroon requires woman-centered, multisectoral strategies that address financial barriers, promote respectful maternity care, and strengthen community-based health education to encourage early initiation and sustained engagement.

## 1 Introduction

Antenatal care (ANC) comprises preventive, promotive, and curative health services provided during pregnancy to optimize maternal and fetal health outcomes through health education, early detection, and management of pregnancy-related complications (1, 2). It remains a cornerstone of maternal and perinatal health, particularly in low- and middle-income countries (LMICs), where maternal morbidity and mortality remain disproportionately high (2, 3). Despite global progress in expanding access to maternal health services, important gaps persist in the adequacy, timing, and quality of ANC utilization.

Effective ANC extends beyond attendance to encompass both the frequency and quality of care, which influence women's satisfaction and continued engagement with services (4). Service quality reflects structural and process dimensions of care, including provider competence, facility conditions, accessibility, and organization. Women's satisfaction represents their subjective evaluation of these dimensions and can shape adherence to medical advice and continuity of care (4–11). In this study, satisfaction is conceptualized both as an outcome of service quality and as a contextual factor influencing continued ANC utilization.

In 2016, the World Health Organization (WHO) revised its ANC guidelines, increasing the recommended minimum from four focused visits to at least eight contacts to enhance maternal and perinatal outcomes through more frequent provider–woman interactions (1). However, uptake of this recommendation has been uneven across LMICs. In sub-Saharan Africa, although approximately 70% of women attend at least one ANC visit, fewer than half complete the recommended number of contacts (3, 7). In Cameroon, fewer than half of pregnant women achieve eight or more ANC contacts, reflecting persistent socioeconomic, cultural, and health system constraints (12).

Previous studies in Cameroon have reported late initiation of ANC, financial barriers, long waiting times, overcrowding, and dissatisfaction with provider attitudes (13–15). However, these studies have largely examined utilization or satisfaction in isolation, with limited integration of women's lived experiences across facility types. In addition, contextual challenges in the South West Region, such as transportation barriers, facility-level resource constraints, and service organization remain underexplored.

This study therefore employed an explanatory sequential mixed-methods design to examine ANC utilization and satisfaction from pregnant women's perspectives across public, private, and faith-based facilities in the Buea and Limbe Health Districts. By integrating quantitative associations with qualitative insights, the study aimed to identify key barriers and facilitators influencing ANC utilization and to examine women's satisfaction with the quality of care received, thereby informing woman-centered and context-responsive interventions.

## 2 Materials and methods

### 2.1 Study design

An explanatory sequential mixed-methods design was employed to examine antenatal care (ANC) utilization and satisfaction among pregnant women. The study consisted of two phases. Phase one involved a facility-based cross-sectional survey to quantify ANC utilization patterns, satisfaction, and associated sociodemographic and service-related factors. Phase two comprised in-depth qualitative interviews to explore contextual, social, and interpersonal factors underlying the quantitative findings.

ANC utilization, defined by completion of the WHO-recommended minimum of eight ANC contacts, was the primary outcome. Overall ANC utilization was assessed using three indicators: timing of the first visit, attendance of the recommended number of visits for the current trimester, and whether any visits were missed. Each indicator was scored 1 for adherence and 0 for non-adherence, giving a maximum score of 3. Women scoring 2 or more (≥60%) were classified as adequately utilizing ANC services, while those scoring less than 2 (<60%) were classified as inadequate (16, 17).

Satisfaction with ANC services was treated as a secondary outcome and as a potential determinant of continued utilization. Integration of quantitative and qualitative findings occurred at the interpretation stage to provide explanatory depth and triangulation.

### 2.2 Study setting

The study was conducted in six health facilities across the Buea and Limbe Health Districts in the South West Region of Cameroon. Facilities were selected to represent public, private, and faith-based sectors. Buea serves as the regional administrative and referral center, while Limbe is an urban coastal district with diverse healthcare providers. Including both districts enabled examination of ANC experiences across different service delivery contexts.

Across facilities, ANC services are typically delivered by obstetricians, midwives and nurses, with variable staffing levels depending on facility ownership and patient volume. Public facilities generally experience higher client loads and longer waiting times, while faith-based and private facilities tend to serve fewer clients but at higher direct costs to users. Referral pathways follow the national health system structure, with complicated pregnancies referred from peripheral health centers to district or regional hospitals.

### 2.3 Study period

Data collection took place over a five-month period from December 2024 to April 2025.

### 2.4 Study population and sampling

The study population comprised pregnant women attending ANC services at selected facilities. Eligibility criteria included residency in the district for at least six months and ability to communicate in English, French, or Pidgin English. Women unable to provide informed consent were excluded.

A multistage sampling approach was used. Facilities were first stratified by ownership type. Within each facility, simple random sampling was conducted using daily ANC attendance lists as the sampling frame. Eligible women were assigned numbers and selected using random number generation during clinic days.

The sample size was calculated using Cochran's formula for cross-sectional studies, assuming a 95% confidence level, 5% margin of error, and a conservative estimated prevalence of 50%, which was chosen due to the absence of prior local estimates of adequate ANC utilization (18). The calculated minimum sample of 384 was increased to 410 to account for potential non-response. As the study was facility-based, women who did not attend ANC were not represented, which may limit generalizability and was acknowledged as a potential source of selection bias.

### 2.5 Data collection procedures

#### 2.5.1 Quantitative data collection

A structured interviewer-administered questionnaire was developed based on a review of validated ANC utilization and satisfaction tools. The questionnaire captured sociodemographic characteristics, obstetric history, knowledge of ANC, timing and frequency of visits, barriers and facilitators to access, and satisfaction with ANC services.

Satisfaction was measured using a five-point Likert scale across multiple service domains. The instrument was pretested among 30 pregnant women in a non-study facility, resulting in minor revisions for clarity and cultural relevance. Internal consistency of the satisfaction scale was acceptable (Cronbach's alpha = 0.82) (19, 20). Trained research assistants conducted face-to-face interviews.

#### 2.5.2 Qualitative data collection

Following preliminary quantitative analysis, 20 participants were purposively selected to capture variation in ANC utilization patterns, facility type, geographic location, and satisfaction levels. Semi-structured interview guides were informed by quantitative findings and explored perceptions of care quality, provider interactions, decision-making processes, cultural influences, and recommendations for improvement. Interviews were audio-recorded with consent and supplemented by field notes. Interviews were conducted until thematic saturation was reached and this was achieved after 20 interviews.

### 2.6 Data analysis and mixed-Methods integration

#### 2.6.1 Quantitative analysis

Quantitative data were analyzed using SPSS version 26. Descriptive statistics summarized participant characteristics and ANC utilization patterns. Bivariate associations were examined using chi-square tests. Multivariable logistic regression was conducted to identify predictors of adequate ANC utilization (≥8 contacts, early ANC initiation and/or Missed ANC composite scores). Variables were selected *a priori* based on literature and conceptual relevance. Multicollinearity was assessed prior to model fitting, and statistical significance was set at *p* < 0.05.

#### 2.6.2 Qualitative analysis

Audio recordings were transcribed verbatim and translated into English where necessary. Data were analyzed thematically using an inductive approach through Dedoose software. Coding was conducted iteratively, with codes refined and grouped into broader themes. Analytical rigor was enhanced through peer debriefing, maintenance of an audit trail, and reflexive memo- writing.

#### 2.6.3 Mixed-methods integration

Integration occurred primarily at the interpretation stage using side-by-side comparison (19). Quantitative findings informed qualitative sampling and interview guide development, consistent with the explanatory sequential design. Joint narrative displays were used to identify convergence, complementarity, and divergence between data strands.

### 2.7 Ethical considerations

Ethical approval was obtained from the Institutional Review Board of the Faculty of Health Sciences, University of Buea. Administrative authorization was granted by relevant health authorities and facility heads. Written informed consent was obtained from all participants. Pregnant adolescents provided assent in line with national IRB guidance for emancipated minors. Confidentiality was maintained using anonymized identifiers, and audio recordings were deleted after transcription.

### 2.8 Artificial intelligence use statement

Artificial intelligence was used solely for language refinement, including grammar and sentence clarity. AI tools did not influence study design, data collection, analysis, or interpretation. The manuscript was carefully reviewed to remove formatting artifacts and ensure consistency.

## 3 Results

### 3.1 Participant recruitment and characteristics

Of the 512 pregnant women approached across six health facilities, 97 declined participation, primarily due to time constraints or lack of interest. Of the 415 women who consented, five were excluded because of incomplete responses, resulting in a final analytic sample of 410 participants, as summarized in Table 1. The mean age of participants was 27.6 ± 5.1 years, with more than half (54.9%) aged between 26 and 35 years. The majority were married (60.5%), Christian (94.9%), and had attained tertiary education (48.3%). Nearly half of the participants were self-employed (49.3%), and 49.5% reported a monthly income below 50,000 FCFA (∼USD 83). Most participants had one or two prior pregnancies (63.4%) and were in their third trimester at the time of interview (53.7%). A substantial proportion (83.2%) reported no complications during the current pregnancy. Participants were distributed across public (49.5%), faith-based (40.5%), and private (10.0%) health facilities.

**Table 1:** Sociodemographic and obstetric characteristics of study participants (*N* = 410).

Variable	Category	Frequency (n)	Percentage (%)
Health facility	Public	203	49.5
	Faith-based	166	40.5
	Private	41	10.0
Age group (years)	18–25	157	38.3
	26–35	225	54.9
	36–50	28	6.8
Marital status	Cohabiting	47	11.5
	Divorced/Separated	4	1.0
	Married	248	60.5
	Single	111	27.0
Religion	Christian	389	94.9
	Muslim	15	3.7
	Traditional	6	1.5
Level of education	No formal education	10	2.4
	Primary	23	5.6
	Secondary	179	43.7
	Tertiary	198	48.3
Occupation	Government employed	27	6.6
	Private employed	52	12.7
	Self-employed	202	49.3
	Student	72	17.6
	Unemployed	57	13.8
Monthly income (FCFA)	<50,000	203	49.5
	50,001–100,000	121	29.5
	100,001–200,000	64	15.6
	>200,000	22	5.4
Number of pregnancies	1–2	260	63.4
	3–4	126	30.7
	5–7	24	5.9
Parity (live births)	No child	69	16.8
	1–2	198	48.3
	3–6	43	10.5
Current trimester	First	19	4.6
	Second	171	41.7
	Third	220	53.7
Pregnancy complications	No	341	83.2
	Yes	69	16.8

### 3.2 Antenatal care (ANC) utilization patterns

Quantitative findings indicated that 45.6% of participants initiated ANC within the first trimester (≤12 weeks), while 52.7% initiated during the second trimester and 1.7% in the third trimester (Table 2). Although early initiation was slightly more frequent in public facilities (50.8%), the association between facility type and timing of first ANC visit was not statistically significant (*χ*^2^ = 12.2, *p* = 0.092). Regarding visit frequency, most women attended between one and three visits (56.8%), followed by four to six visits (37.1%), while only 6.1% (25/410) attended seven to ten visits, achieving the WHO-recommended minimum of eight contacts (Table 2). Facility type was significantly associated with visit frequency (*χ*^2^ = 33.45, *p* < 0.001), with faith-based facilities accounting for the highest proportion of women attending seven to ten visits. Most participants (87.3%; 358/410) reported no missed ANC visits, and among those who missed appointments (12.7%; 52/410), missed visits were significantly associated with facility type (*χ*^2^ = 25.35, *p* = 0.045), with over half occurring among users of faith-based facilities.

**Table 2:** Antenatal care utilization by type of health facility (*N* = 410).

Variable	Category	Public n (%)	Faith-based n (%)	Private n (%)	Total n (%)	*χ* ^2^	*p*-value
First ANC visit (weeks)	0–12	95 (50.8)	73 (39.0)	19 (10.2)	187 (45.6)	12.20	0.092
	13–27	105 (48.7)	90 (41.6)	21 (9.7)	216 (52.7)		
	28–40	3 (42.9)	3 (42.9)	1 (14.2)	7 (1.7)		
Number of ANC visits	1–3	119 (51.1)	90 (38.6)	24 (10.3)	233 (56.8)	33.45	<0.001
	4–6	76 (50.0)	61 (40.1)	15 (9.9)	152 (37.1)		
	7–10	8 (32.0)	15 (60.0)	2 (8.0)	25 (6.1)		
Missed ANC visits	No	179 (50.0)	139 (38.9)	40 (11.1)	358 (87.3)	25.35	0.045
	Yes	24 (46.2)	27 (51.9)	1 (1.9)	52 (12.7)		

Percentages represent row-wise distribution.

Qualitative insights complemented these findings. Women reported attending ANC primarily for fetal wellbeing, access to supplements, and health education, with one participant noting, “Each time I go, I learn something new… I feel like they're preparing me for the baby” (Participant 6, Limbe RH – public facility). Barriers to consistent attendance included long waiting times, drug stock-outs, staff absenteeism, negative provider attitudes, limited partner support, and reluctance to disclose pregnancy early, as illustrated by one participant: “Because I'm not married… they talk to me harshly” (Participant 1, Buea RH – public facility).

### 3.3 Factors associated with adequate ANC utilization

Multivariable logistic regression results are presented in Table 3, examining factors associated with adequate ANC utilization (≥8 visits versus <8 visits). After adjusting for sociodemographic and obstetric characteristics, facility type, employment status, parity, and timing of ANC initiation remained significantly associated with achieving the recommended number of visits. Specifically, women attending public health facilities, those formally employed, women with one or two children, and those initiating ANC in the second trimester had higher odds of completing at least eight visits.

**Table 3:** Multivariable logistic regression analysis of factors associated with adequate ANC utilization (*N* = 410).

Variable	Category	Adjusted OR (95% CI)	*p*-value
Facility type	Public	2.54 (1.12–5.77)	0.026
	Faith-based	2.12 (0.93–4.83)	0.072
	Private (Reference)	1.00	—
Occupation	Private employed	3.18 (1.30–7.82)	0.012
	Government employed	3.13 (1.94–10.48)	0.044
	Self-employed	1.10 (0.54–2.22)	0.803
	Student	1.92 (0.83–4.42)	0.125
	Unemployed (Reference)	1.00	—
Parity	1–2 children	2.00 (1.00–4.48)	0.041
	No child	1.40 (0.84–2.35)	0.198
	≥3 children (Reference)	1.00	—
Trimester at booking	Second trimester	8.75 (2.74–27.97)	<0.001
	First trimester	1.48 (0.46–4.72)	0.509
	Third trimester (Reference)	1.00	—
Monthly income (FCFA)	>200,000	0.69 (0.23–2.04)	0.496
	100,001–200,000	0.67 (0.34–1.32)	0.248
	50,000–100,000	0.92 (0.53–1.60)	0.772
	<50,000 (Reference)	1.00	—

Adequate ANC utilization was defined using a composite score derived from three indicators: timing of the first ANC visit, attendance of recommended visits for the current trimester, and missed ANC visits (adequate ≥2 indicators met). Adjusted odds ratios were obtained from a multivariable logistic regression controlling for selected sociodemographic and obstetric variables.

### 3.4 Qualitative corroboration

Qualitative findings reinforced the quantitative results, illustrating how financial capacity and respectful provider behavior facilitated continued engagement. Participants noted that employment enabled affordability of transport and medical services, while respectful care motivated ongoing attendance. One participant explained, “We should not be made to sit so long… I almost stopped going” (Participant 12, Ambition – private facility).

### 3.5 Satisfaction with ANC services

Analysis of satisfaction ratings, summarized in Table 4, indicated that overall satisfaction with ANC services was high, particularly with respect to cleanliness, staff attitude, and perceived quality of care. However, waiting times and the cost of services were the main sources of dissatisfaction. Qualitative narratives highlighted the centrality of provider interactions to satisfaction. Positive experiences, such as attentive communication and empathy, enhanced satisfaction, exemplified by the statement: “She smiled… asked how I was feeling” (Participant 5, Mount Mary - faith-based facility). Dissatisfaction arose from long queues, lack of privacy, and additional out-of-pocket expenditures, as one participant described: “You pay… then buy drugs outside” (Participant 11, Buea RH).

**Table 4:** Satisfaction with antenatal care (ANC) service domains among pregnant women (*N* = 410).

Service Domain	% Satisfied^a^	% Dissatisfied^b^
Waiting time	39.2	32.2
Staff attitude	73.2	10.5
Privacy during consultation	67.8	14.3
Cleanliness of facility	78.3	8.1
Availability of equipment	67.1	8.0
Information provided	70.7	10.7
Cost of services	59.5	16.8
Provider communication	71.2	10.8
Overall quality of care	75.4	9.8

^a^ Satisfied = satisfied + very satisfied.

^b^ Dissatisfied = dissatisfied + very dissatisfied.

### 3.6 Integrated summary of key findings

Integration of quantitative and qualitative findings, summarized in Tables 5, 6, revealed that second-trimester ANC initiation was statistically associated with adequate utilization, although qualitative data showed that early initiation was often delayed by cultural beliefs, fear of stigma, and limited awareness. Facility type, employment, and provider behavior consistently influenced both utilization and satisfaction. While overall satisfaction remained high, persistent challenges, including waiting times, provider attitudes, and indirect costs, continued to undermine engagement with care.

**Table 5:** Qualitative findings organized in themes, subthemes and participants' quotes.

Theme	Subtheme	Key Insights	Illustrative Quotes
1. Motivation for Attending ANC	Health Education & Fetal Wellbeing	Women attended ANC to monitor their baby'shealth, receive advice, and access supplements	“Each time I go, I learn something new… I feel like they're preparing me for the baby.” – Participant 6, Limbe RH (public facility)
	Social Influence	Encouragement from peers and family fostered attendance.	“My neighbor is also pregnant and she encouraged me. We sometimes go together.” – *Participant 2,* *Limbe RH*
2. Barriers to ANC Attendance	Service Inefficiencies	Long waiting times, unavailable drugs, and staff absenteeism discouraged visits.	“Sometimes… they say the nurse is not around… it's very frustrating.” – *Participant 2, Limbe RH*
	Poor Staff Attitudes	Negative or disrespectful behavior from providers made women feel humiliated or unwelcome.	“One day I came wearing slippers… the nurse said, ‘Next time dress properly.’’ – Participant 10, Buea RH (public hospital)
	Financial Constraints	Transport and out-of- pocket costs limited access.	“You pay 1000 francs… then buy drugs outside… I've spent over 4000 francs.’’ – *Participant 11,* *Buea RH*
	Transportation & Distance	Long distances, bad roads, and expensive bike rides made it difficult to attend.	“If it rains or I don't have money, I just miss the appointment.’’ – Participant 8, Mount Mary (faith- based facility)
	Cultural Beliefs	Traditional views discouraged early ANC.	“They advise us to wait until the belly is big… I don't listen to that anymore.’’ – Participant 7, Solidarity (private facility)
	Lack of Family Support	Absence of partner/family support reduced ANC visits.	“The man responsible doesn't care… sometimes I just stay home.’’ – Participant 9, Solidarity(private)
3. Satisfaction with ANC Services	Provider Interactions	Respectful, caring staff improved satisfaction.	“She smiled… asked how I was feeling… I felt like I had someone walking with me.’’ – Participant 5, Mount Mary(faith-based)
	Disrespect & Judgment	Shouting or harsh comments, especially towards unmarried women, were common.	“Because I'm not married… they talk to me harshly. One even said, ‘Use protection next time.’’ – *Participant 1, Buea RH*
	Waiting Time	Long queues and delayed services caused stress and discomfort.	“We should not be made to sit so long… I almost stopped going.’’ – Participant 12, Ambition(private facility)
4. Quality of Information & Counseling	Clarity & Comprehension	Women valued clear explanations about health status and care plans.	“They explained my blood pressure… I wasn't scared anymore because I knew what to do.’’ – *Participant 6, Limbe RH*
	Insufficient Health Education	Gaps in birth preparation and danger sign education were noted.	“No one talks about what to do if your water breaks… we need more than just drugs.’’ – Participant 7, Solidarity (private facility)
	Communication Style	Participants preferred interactive, simplified communication.	“They should use pictures or charts… not everyone can follow fast.’’ – *Participant 1, Buea RH*
5. Key Drivers for Facility Choice & Recommendations	Environmental & Interpersonal Factors	Cleanliness, staff behavior, and equipment availability influenced choice.	“I chose the facility because it's clean and the staff are friendly.’’ (General response)
6. Suggested Improvements	Service Delivery	Increase staff, reduce waiting times, and ensure drug availability.	“They should make the process faster. We are not supposed to sit all day while pregnant.’’ – Participant 10, Buea RH(public facility)
	Empathy & Respect	Improve provider- patient communication and empathy.	“Even just a kind word can make a big difference.’’ – Participant 10, Buea RH(public facility)

**Table 6:** Summary of integration of key quantitative and qualitative findings.

Key Area	Quantitative Evidence	Qualitative Insight
Timing of initiation	Second trimester start significantly associated with adequate use (*p* < 0.001)	Early ANC delayed due to cultural beliefs, stigma, and unawareness
Facility type	Public facilities linked to better ANC uptake (*p* = 0.026)	Choice influenced by cleanliness, familiarity, and perceived respect
Barriers to access	Missed visits associated with facility type (*p* = 0.045)	Long waiting times, transport costs, and poor provider behavior discouraged use
Satisfaction	75.4% satisfied with overall care; low satisfaction with waiting times	Friendly, respectful staff enhanced trust; judgment and long waits reduced it
Employment	Employed women had significantly higher odds of adequate ANC use (*p* < 0.05)	Stable income facilitated transport and payment for services and medications

## 4 Discussion

### 4.1 Overview of key findings and mixed-methods integration

This mixed-methods study examined antenatal care utilization and satisfaction among pregnant women in the Buea and Limbe Health Districts, integrating quantitative and qualitative findings to explain not only what influenced ANC use but why. The results indicate that structural, interpersonal, and cultural factors concurrently shape women's engagement with ANC services. By integrating women's narratives with statistical associations, the study provides contextualized explanations for observed utilization and satisfaction patterns.

### 4.2 Socioeconomic and demographic determinants of ANC utilization

Adequate ANC utilization, defined as attending at least eight contacts, was achieved by only 52.2% of participants, a proportion lower than national estimates and some regional reports (13, 21). This gap highlights the persistent challenge of translating ANC availability into consistent, complete use. Employment emerged as a significant facilitator of adequate utilization, as formally employed women were better able to afford transport, laboratory tests, and medications, consistent with findings from Nigeria and Ghana (5, 22, 23).

Marital status also influenced utilization, with married women attending more regularly, likely due to partner support both financially and emotionally. In contrast, unmarried women reported stigma and discouraging provider behaviors, which negatively affected their engagement with services. These findings are consistent with reports from Ghana, Namibia, and Indonesia, where social norms and provider attitudes similarly constrained care-seeking among unmarried pregnant women (24, 25).

### 4.3 Timing of ANC initiation and continuity of care

Interestingly, second-trimester initiation was associated with higher odds of completing the recommended number of ANC contacts, a finding that is counterintuitive relative to WHO guidance recommending first-trimester booking. This association should be interpreted cautiously and should not be construed as endorsing delayed initiation. Qualitative findings revealed that early initiation was often postponed due to cultural beliefs, fear of pregnancy loss, stigma, and limited awareness of the benefits of early ANC.

Women who initiated care in the second trimester may have had more stable circumstances or greater motivation to complete follow-up visits once care began, resulting in higher cumulative contact attendance. Similar patterns have been reported in Ethiopia and other Cameroonian settings, where late initiation did not necessarily preclude continuity of care once engagement was established (14, 26).

### 4.4 Facility type, parity, and trade-offs in care-seeking

Parity and facility type further influenced ANC utilization. Women with one or two children were more likely to achieve recommended contacts, possibly reflecting retained health knowledge without the complacency sometimes observed among multiparous women. Public facilities were associated with higher utilization, likely due to lower costs, broader service availability, and perceived reliability (15).

However, qualitative findings revealed important trade-offs. Public facilities were often described as efficient but characterized by long waiting times and limited privacy, while faith- based facilities were perceived as more empathetic and respectful but experienced higher rates of missed visits due to cost and scheduling constraints. These findings underscore that utilization patterns do not necessarily equate to perceived quality.

### 4.5 Barriers and facilitators to ANC utilization

Key barriers to ANC use included financial constraints, transport challenges, and knowledge gaps regarding the recommended number of ANC contacts. Only 28.5% of participants were aware of the WHO eight-contact guideline, and misconceptions about early pregnancy care contributed to delayed initiation (6, 23, 27–29).

Conversely, facilitators included respectful provider behavior, partner support, and clear, consistent health education. Women emphasized that empathy, dignity, and nonjudgmental communication from providers motivated continued attendance, highlighting the central role of respectful maternity care in sustaining utilization and shaping satisfaction.

### 4.6 Satisfaction with ANC services and provider behavior

Overall satisfaction with ANC services was high (75.4%), particularly regarding cleanliness and staff attitude, aligning with evidence from East Africa (28–31). Nonetheless, dissatisfaction related to waiting times, privacy, and perceived poor value for money pointed to systemic inefficiencies within service delivery.

**Figure 1:**
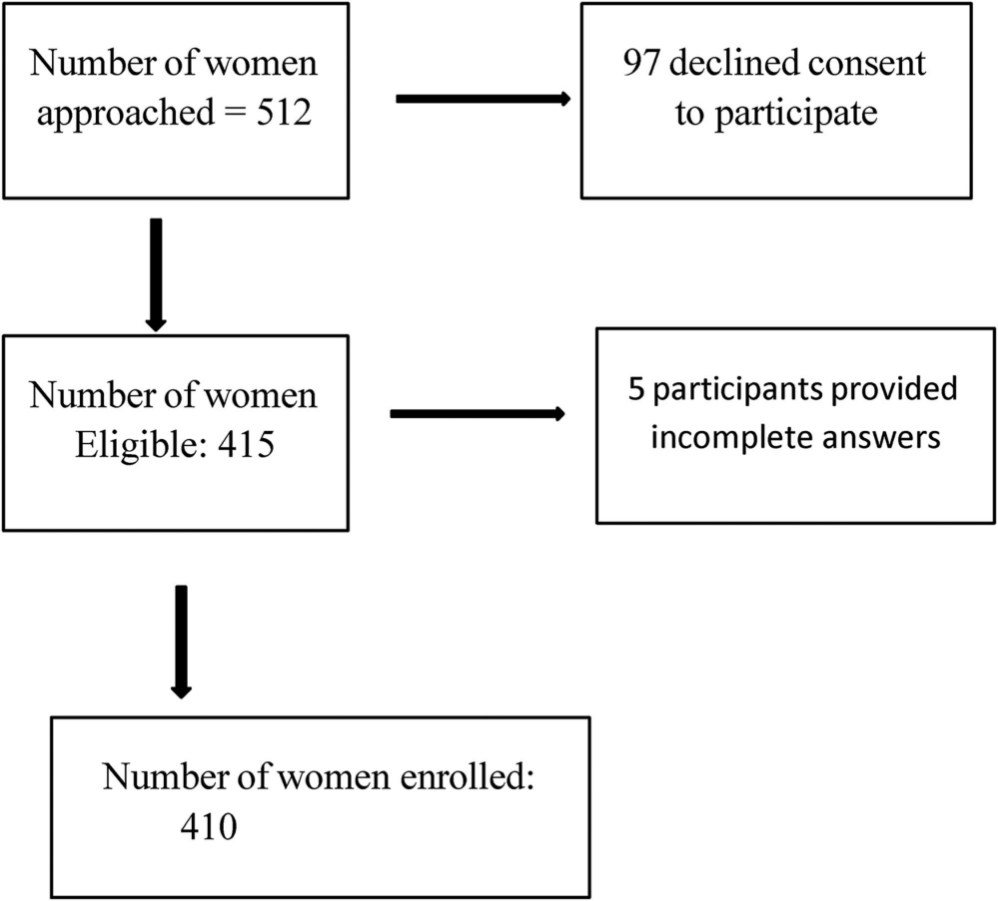
Participants' flow diagram.

Provider behavior emerged as a central determinant of satisfaction. Judgmental interactions, especially toward unmarried women, undermined trust and discouraged continued engagement, whereas respectful communication fostered confidence and sustained attendance. These findings reinforce existing evidence that interpersonal quality of care is as critical as technical competence in maternal health services (32, 33).

### 4.7 Strengths and limitations

This study has several strengths, including its mixed-methods design, the inclusion of diverse facility types, and the integration of women's perspectives, which provide contextually grounded insights relevant for policy and practice in similar settings. The explanatory sequential approach enhanced interpretation by allowing qualitative findings to contextualize quantitative associations.

However, several limitations should be acknowledged. Data were self-reported and may be subject to recall and social desirability bias. As the study was facility-based, women who did not attend antenatal care (ANC) were not represented; this may have biased the sample toward women with relatively better access to health services, higher motivation, or stronger health-seeking behavior, thereby limiting generalizability to all pregnant women in the study area. In addition, the cross-sectional design precludes causal inference. Finally, facility-level characteristics such as staffing levels, provider workload, and drug availability were not directly measured and may have influenced both ANC utilization and satisfaction outcomes.

## 5 Conclusion and recommendations

Pregnant women in Buea and Limbe generally reported positive experiences with antenatal care (ANC) services; however, adequate utilization remains suboptimal. Both ANC utilization and satisfaction were shaped by health system factors, socio-economic conditions, provider behavior, and community norms. Respectful care, financial capacity, partner support, and effective communication were central to sustaining ANC engagement, while delayed initiation largely reflected structural and cultural barriers rather than informed choice.

To address these challenges, a woman-centered and multisectoral approach is recommended. Financial barriers to care should be reduced through progress toward universal health coverage and targeted cost-mitigation strategies. Community-based health education should emphasize the importance of early first-trimester ANC initiation. Within health facilities, strengthening respectful maternity care through training in nonjudgmental communication, empathy, and client-centered practices is essential for improving both satisfaction and continuity of care. Service organization should also be optimized to reduce waiting times and indirect costs. Within this framework of respectful and well-organized care, male and family involvement should be actively encouraged as part of routine ANC services to enhance emotional, logistical, and financial support for pregnant women. Collectively, these strategies can improve both the utilization and quality of ANC services and contribute to progress toward Sustainable Development Goal 3.

## Data Availability

The data analyzed in this study is subject to the following licenses/restrictions: Patient identity protection. Data available upon reasonable request from corresponding author Requests to access these datasets should be directed to rtchounzou@yahoo.fr.
